# Restricted-Range Fishes and the Conservation of Brazilian Freshwaters

**DOI:** 10.1371/journal.pone.0011390

**Published:** 2010-06-30

**Authors:** Cristiano Nogueira, Paulo A. Buckup, Naercio A. Menezes, Osvaldo T. Oyakawa, Thais P. Kasecker, Mario B. Ramos Neto, José Maria C. da Silva

**Affiliations:** 1 Programa Cerrado-Pantanal, Conservação Internacional do Brasil, Brasília, Distrito Federal, Brazil; 2 Departamento de Vertebrados, Museu Nacional, Universidade Federal do Rio de Janeiro, Rio de Janeiro, Rio de Janeiro, Brazil; 3 Seção de Ictiologia, Museu de Zoologia, Universidade de São Paulo, São Paulo, São Paulo, Brazil; 4 Programa Amazônia, Conservação Internacional do Brasil, Belém, Pará, Brazil; Smithsonian's National Zoological Park, United States of America

## Abstract

**Background:**

Freshwaters are the most threatened ecosystems on earth. Although recent assessments provide data on global priority regions for freshwater conservation, local scale priorities remain unknown. Refining the scale of global biodiversity assessments (both at terrestrial and freshwater realms) and translating these into conservation priorities on the ground remains a major challenge to biodiversity science, and depends directly on species occurrence data of high taxonomic and geographic resolution. Brazil harbors the richest freshwater ichthyofauna in the world, but knowledge on endemic areas and conservation in Brazilian rivers is still scarce.

**Methodology/Principal Findings:**

Using data on environmental threats and revised species distribution data we detect and delineate 540 small watershed areas harboring 819 restricted-range fishes in Brazil. Many of these areas are already highly threatened, as 159 (29%) watersheds have lost more than 70% of their original vegetation cover, and only 141 (26%) show significant overlap with formally protected areas or indigenous lands. We detected 220 (40%) critical watersheds overlapping hydroelectric dams or showing both poor formal protection and widespread habitat loss; these sites harbor 344 endemic fish species that may face extinction if no conservation action is in place in the near future.

**Conclusions/Significance:**

We provide the first analysis of site-scale conservation priorities in the richest freshwater ecosystems of the globe. Our results corroborate the hypothesis that freshwater biodiversity has been neglected in former conservation assessments. The study provides a simple and straightforward method for detecting freshwater priority areas based on endemism and threat, and represents a starting point for integrating freshwater and terrestrial conservation in representative and biogeographically consistent site-scale conservation strategies, that may be scaled-up following naturally linked drainage systems. Proper management (e. g. forestry code enforcement, landscape planning) and conservation (e. g. formal protection) of the 540 watersheds detected herein will be decisive in avoiding species extinction in the richest aquatic ecosystems on the planet.

## Introduction

Freshwaters are the most threatened ecosystems on earth [1], [2], [3]. Although recent global-scale biodiversity assessments provide important data on priority regions for freshwater conservation [2], local watershed-scale priorities remain poorly known for most drainage systems on the planet, hampering effective and focused local action. Refining the scale of global biodiversity assessments (both at terrestrial and freshwater realms) and translating these into conservation priorities on the ground remains a major challenge to biodiversity science [4], [5], and depends directly on species occurrence data with fine taxonomic and geographic resolution [2], [6], [7].

Apart from lack of basic data, effective implementation of local scale conservation actions are also hampered by the fact that most conservation assessments tend to treat terrestrial and freshwater ecosystems as independent ecological and biogeographical units [1], [8], [9]. This lack of integration neglects the interdependence between terrestrial and freshwater ecosystems, and favors a bias towards better known terrestrial systems and organisms (especially endothermic vertebrates), while freshwater biodiversity remains neglected in most priority setting analyses [1], [2], [3], [8], [10].

Fishes are the most studied group and the best indicators of zoogeographical patterns among obligate aquatic taxa [2]. Owing to dispersal limitations not found among terrestrial organisms, many freshwater fish species have relatively localized distributions [11], [12]. Due to high levels of endemism and human pressure, freshwater fish faunas around the world are under serious threats [12]. Threats to freshwater fish species require special attention because historical influences on distribution and diversity patterns may be more evident in freshwater fishes than in other taxonomic groups, and detailed patterns of endemism and distribution of freshwater fishes differ from those in birds and mammals [11], the two best studied vertebrate groups in terms of threats and conservation priorities [13].

Brazil harbors the world's richest freshwater ichthyofauna [14], which remains far from being completely documented and studied, despite the recent acceleration of discovery and description of new fish species. Over 267 freshwater fish species have been described from 2001 to 2005, and Brazilian ichthyology is currently experiencing its most productive period [15], [16]. While knowledge accumulates, species extinctions in Brazilian freshwaters are already being documented, and many species may be under serious threat even before being formally described or studied in basic aspects of natural history [16]. Despite extraordinarily high diversity and growing threats from river impoundment projects, water siltation and pollution, and riparian habitat destruction through deforestation, agriculture and urban growth, no comprehensive conservation analysis has ever been conducted on Brazilian freshwater fishes [16].

Herein we provide the first detailed assessment of site-scale freshwater conservation priorities in the Neotropical Region, using validated occurrence data from a comprehensive set of fishes with restricted geographic distribution and information on threats in Brazilian river systems. The main goals of our study are to detect and delineate catchment areas harboring narrow ranging endemic freshwater fishes, highlighting critical areas for avoiding extinction in the worlds richest freshwater ecosystems.

## Methods

In the first step of the study we gathered and analyzed raw distribution data in museums and taxonomic literature in order to select restricted-range fish species from a complete list of freshwater fishes known to occur in Brazil in the beginning of 2007 [16], [18]. The main data source was the recently published “Catálogo das Espécies de Peixes de Água Doce do Brasil”[16]. This catalogue represents an exhaustive compilation of taxonomic and distributional data for 2587 species of the Brazilian freshwater ichthyofauna, and involved the collaboration of 39 ichthyologists, from most major institutions actively involved in studying Brazilian freshwater fish diversity[16]. These icthyologists were selected based on their expertise as specialists in the various taxonomic groups and their ability to produce a timely revision of reliable species distribution data. All major museums in Brazil and abroad were the source of taxonomic and distributional data, coupled with an extensive list of primary literature data (including more than 540 cited references). In addition to validating the taxonomic limits of all species with demonstrated occurrence in Brazil, the 39 authors were assigned the task of establishing the geographic distribution range of each species based only on voucher specimen-based data or state-of-the art taxonomic revisions. Unverifiable records were discarded from the analyses [16].

In a second step, we reviewed the 2.587 species distributions thus generated, and selected a subset of restricted-range taxa according to the criteria set forth below. The stated distributions in this reduced subset were then reviewed based on actual locality data available from reliable museum locality records and primary literature data. These locality records were then validated on a case-by-case confrontation with spatial data available from topographic maps and gazetteers, using DIVA-GIS software [17]. Taxa with imprecise locality data were discarded and distribution data corrected as necessary.

Because of the fine-grained distributions of fishes [4], [11], we defined as restricted-range species those with known distributions not exceeding 10.000 km^2^, a conservative adaptation of the larger threshold of 50.000 km^2^ pioneered for birds [18]. Species known only from poorly defined localities were excluded.

In a third step, we associated each restricted-range species to a point locality according to a georeferenced voucher-based locality record, generally the type-locality of each species. Geographic coordinates were recorded using the most precise information available (usually to the nearest minute). When the exact location of the type-locality was unknown, or when there were indications that the type-populations were extinct, we used more recent voucher-documented data. The main sources for georeferencing localities were coordinates obtained from literature data, gazzetteers [19], [20] and museum records. Museum records were obtained primarily from, but not restricted to, Museu de Zoologia, Universidade de São Paulo (MZUSP), Museu Nacional, Universidade Federal do Rio de Janeiro (MNRJ), and Museu de Ciências e Tecnologia, Pontifícia Universidade Católica do Rio Grande do Sul. Together, these collections comprise the most comprehensive set of large-scale faunal surveys currently available for Brazilian freshwater fishes. In cases where no precise coordinates were available, but the type-locality was described in some detail, coordinates were recovered using traditional and digital mapping procedures [17], by inspection of locality descriptions and available cartographic layers (e.g. river systems, roads, watersheds, topography, municipalities protected areas, see [21]).

In the fourth step, we associated watershed areas to each species according to their respective point locations, using a digital map of Brazilian watersheds [22] constructed according to the Otto Pfafstetter method [23]. This digital map divides Brazilian hydrographic basins into the smallest detectable catchment areas (Ottobasins) in the 1∶1.000.000 scale. These catchment areas are grouped hierarchically from 1^st^ (largest) to 12^th^ order basins [24]. We delineated each site as the first largest hydrographic basin containing the 1∶1.000.000 catchment area of the locality record. In order to obtain hydrographic basins consistent with the proposed uppermost limit of geographic ranges (10.000 km^2^), we delineated watershed areas using Ottobasins ranging from 6th to 4th order. As each of the selected species is known from limited records within a single small-scale watershed, having been recorded nowhere else, each selected catchment area is expected to contain or roughly correspond to the entire distribution of the associated restricted-range species. After delineation, we assigned species and their corresponding watershed areas to Brazilian hydrographic regions [25], [26] and to Brazilian official biomes [27], according to the location of species locality records.

Protection status of each watershed area was assessed according to its intersection with conservation units [28] and indigenous lands [29]. As freshwater ecosystems are directly affected by changes in surrounding terrestrial habitats [30], watershed habitat integrity and quality was estimated based on data on natural vegetation cover [31]. Watersheds were considered under direct impact of hydroelectric dams if containing 6^th^ order (or smaller) Ottobasins coincident with projected or installed hydropower plants of at least 30MW power output, the threshold value for considering large hydropower projects in Brazil [32]. Threat status of each species was assessed according to IUCN criteria [33], using range size and habitat loss information. Critical watersheds were those showing either one of the following conditions: 1) combined intense levels of habitat loss (≤30% original vegetation) and lack of formal protection (≤30% overlap with protected areas); 2) or under direct impact of hydropower dams.

## Results

**Figure 1:**
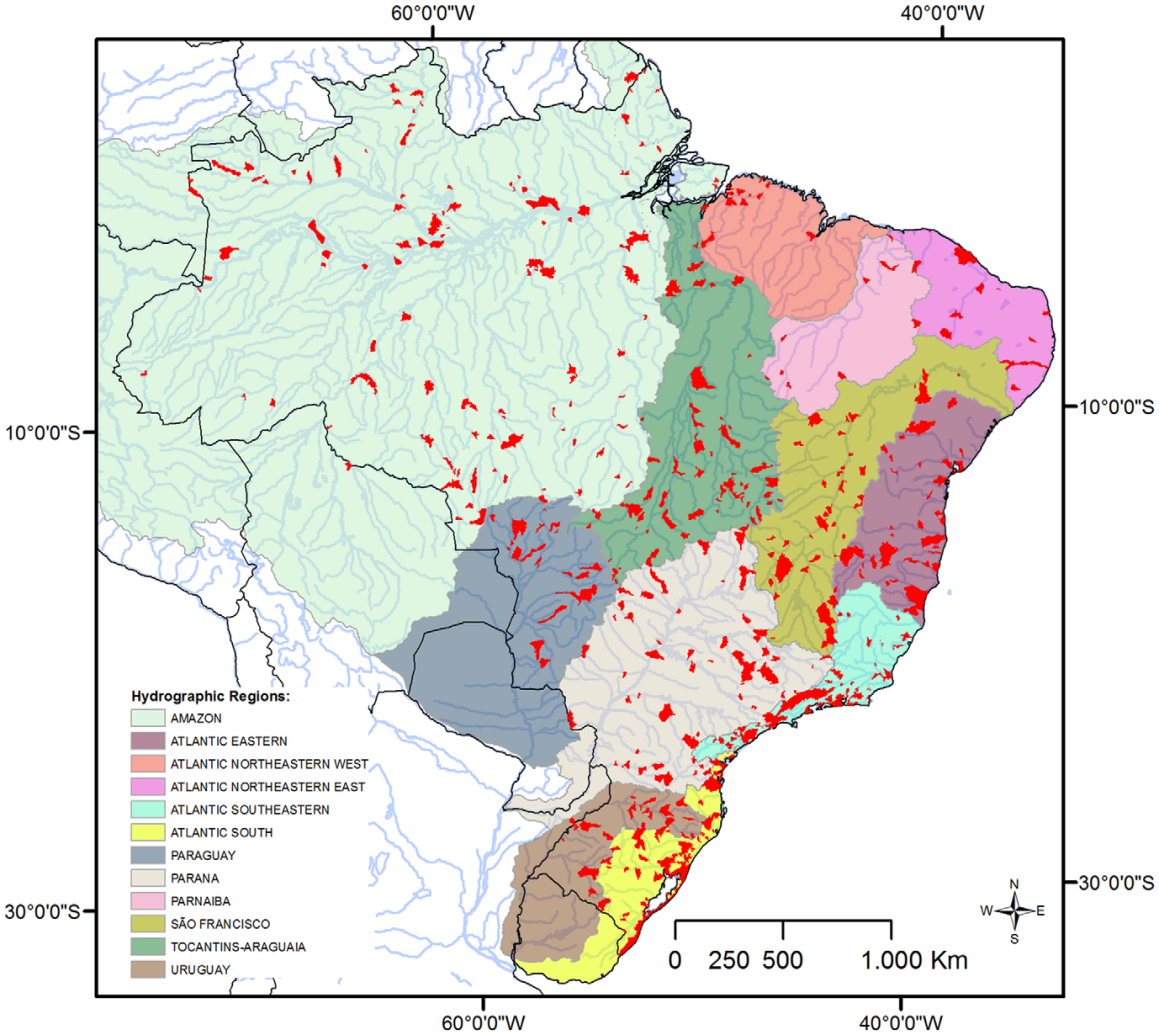
Watersheds containing Brazilian restricted-range freshwater fishes. The 540 small scale watersheds are shown according to Brazilian major hydrographic regions.

A total of 819 fish species with validated information on their distribution were considered as restricted-range species and were included in our study. These comprise ca. 32% of the Brazilian freshwater fish fauna. The geographic distributions of the restricted-range species define 540 small-scale watersheds (Fig. 1), ranging in size from 40 to 9,177 km^2^ (average size 852±1,008 km^2^). Additional data for the 540 watersheds and endemic freshwater fishes is supplied as supporting information (Table S1).

The sum of these watersheds represents an area of 460,301 km^2^ (Table 1), or roughly 5% of the Brazilian territory. These watersheds harbor one to fourteen species each, and are found in all large Brazilian hydrographic regions, but mostly in the Amazon, Tocantins-Araguaia, and Paraná systems (Table 1, Fig. 1). Most watersheds are defined by low numbers of restricted-range species, but some show high correlation between restricted distribution and levels of endemism, having as many as 10 (Paraíba do Sul) or 14 (Iporanga) restricted-range species species. Most of the detected species (530; 65%) are found in watersheds within the Cerrado and Atlantic Forests, terrestrial regions detected as global conservation priorities due to the coincidence of high endemism and habitat loss [34], [35].

**Table 1:** Number of watersheds, number of species, percentage of native vegetation cover, protection status and number of critical watersheds according to the distribution of 819 restricted-range fish species and the 11 Brazilian main hydrographic regions.

Hydrographic region	Watersheds	Species	Average Site area (km^2^)	Average % Natural Cover	% Natural Cover				Average % Protection	Protection				Critical Sites (%)
					≤30	>30<50	≥50<70	≥70		≤30	>30<50	≥50<70	≥70	
Amazon	124	184	815±782	76±26	11	13	16	84	33±41	77	12	2	33	35 (27)
Atlantic Eastern	46	67	1038±1364	50±33	18	7	5	16	18±29	36	5	0	5	21 (46)
Atlantic Northeastern West	11	14	746±552	28±18	5	5	1	0	10±26	10	0	0	1	5 (45)
Atlantic Northeastern East	12	20	1357±1071	46±28	4	0	5	3	9±17	10	2	0	0	3 (25)
Atlantic Southeastern	54	112	594±1239	52±29	13	17	6	18	26±32	35	9	3	7	20 (37)
Atlantic South	59	86	691±670	52±28	15	13	10	21	14±26	49	4	1	5	24 (41)
Paraguay	21	25	1235±1095	65±30	3	1	7	10	21±33	16	1	1	3	4 (19)
Parana	64	99	984±936	24±25	42	9	11	2	18±31	50	2	5	7	50 (78)
Parnaiba	9	15	618±373	80±34	1	0	1	7	41±44	4	1	1	3	0 (0)
São Francisco	41	62	1020±1400	51±34	13	7	6	15	16±31	34	1	1	5	16 (30)
Tocantins-Araguaia	69	101	810±952	67±35	14	7	10	38	22±34	48	6	5	10	22 (32)
Uruguay	30	34	725±678	27±29	20	5	1	4	2±4	30	0	0	0	20 (67)
Total	540	819	852±1008	55±34	159	84	79	218	21±33	399	43	19	79	220 (41)

**Figure 2:**
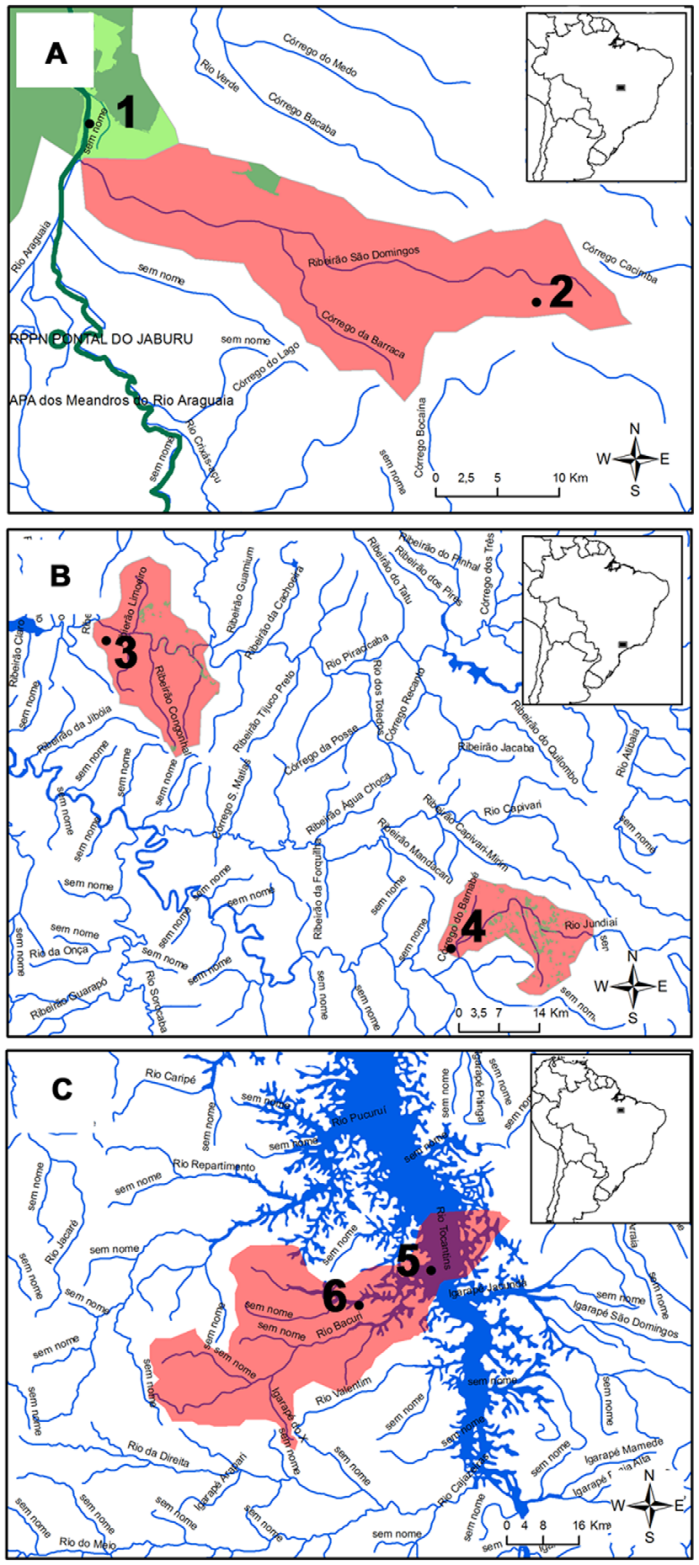
Examples of critical watersheds. (a) Watershed showing less than 30% overlap with protected areas. (b) Watersheds showing less than 30% original habitats. (c) Watersheds under direct impact of hydroelectric dam. Dark green lines indicate protected area boundaries, pale green irregular polygons within detected watersheds indicate terrestrial habitat remnants. Dots indicate available records of restricted-range species. 1: *Curimata acutirostris* Vari & Reis 1995 (Characiformes: Curimatidae); 2: *Melanocharacidium auroradiatum* Costa & Vicente 1995 (Characiformes: Crenuchidae); 3: *Hypostomus paulinus* (Ihering 1905) (Siluriformes: Loricariidae); 4: *Corydoras flaveolus* Ihering 1911 (Siluriformes: Callichthydae); 5: *Harttia duriventris* Rapp Py-Daniel & Oliveira 2001 (Siluriformes: Loricariidae); *Typhlobelus macromycterus* Costa & Bockmann 1994 (Siluriformes: Trichomycteridae); 6: *Mylesinus paucisquamatus* Jégu & Santos 1998 (Characiformes: Characidae).

**Figure 3:**
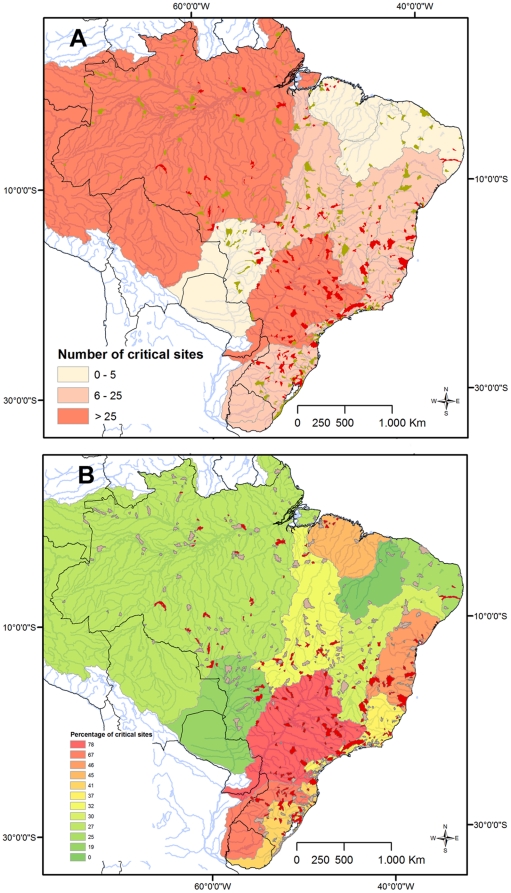
Critical watersheds in major Brazilian hydrographic regions. Classification of Brazilian hydrographic regions according to (a) number and (b) percentage of critical watersheds. Critical watersheds (sites under direct impact of hydropower plants, or under combined poor formal protection and high rates of habitat loss) are marked in red.

Most of the watersheds (399;74%) harboring restricted-range species have less than 30% of their area formally protected (Table 1, see example in Fig. 2a). Extremely low levels of habitat integrity (≤30% original vegetation) were detected in 159 (29%) watersheds (Table 1, see example in Fig. 2b). One hundred and twenty (22%) watersheds are under direct impact of hydropower plants (see example in Fig. 2c), and harbor 220 (27%) endemic fishes. Combining threats, we detected 220 critical watersheds overlapping hydroelectric dams (see Fig. 2c) or showing both poor formal protection and widespread habitat loss (see Fig. 2a); these critical sites harbor 344 fish species that may face extinction if conservation action is not implemented in the near future. The Paraná Hydrographic Region contains the highest number (50) of critical watersheds. Other areas with high numbers of critical sites include the Amazon (35), Atlantic South (24), Tocantins-Araguaia (22), Atlantic Eastern (21), Uruguay, Atlantic Southeastern (20), and São Francisco (16) hydrographic regions (Table 1; Fig. 3a). When the total number of watersheds with restricted-range species is considered, the Amazon, Parnaíba, Atlantic Northeastern, Atlantic Eastern, and Paraguay regions show low proportions of critical watersheds (less than 30% of total), with highest percentages found in Paraná (78%) and Uruguay (67%) regions (Fig. 3b). Most species found in critical watersheds are recorded within the Atlantic Forest and Cerrado regions, previously detected as global conservation priorities. However, at the local scale, roughly one third of the watersheds (164 of 540) show poor area overlap (≤30%) with Brazilian priority areas for conservation [36].

Dominant genera among restricted-range species were *Trichomycterus* (Siluriformes: Trichomycteridae) with 45 species, and *Simpsonichthys* (Cyprinodontiformes: Rivulidae), with 43 species. All seven globally threatened fish species in Brazil [37], and 106 (80%) of the 133 Brazilian fishes in the Brazilian official redlist [38] showed restricted ranges and are included in our analyses. However, 679 (83%) restricted-range species were never evaluated in global or regional threat assessments. By using IUCN's criteria [33] applied to range sizes and habitat integrity, all 819 detected restricted-range species could be classified as threatened. Assuming that species in areas with less than 70% original vegetation cover face continuing decline in extent and quality of habitat, 11 species (1%) restricted to watersheds with less than 100 km^2^ would be considered critically endangered (under criterion B1a, b i;iii). Using the same criterion, other 449 (55%) species found in watersheds smaller than 5.000 km^2^ would be classified as endangered, and 22 species found in areas smaller than 20.000 km^2^ would be classified as vulnerable. The remaining 337 species, despite their occurrence in areas under less intense rates of habitat loss, would be classified as vulnerable, being known from less than five locations and susceptible to the effects of human activities or stochastic events in an uncertain future, and being thus capable of becoming critically endangered or even extinct in a very short time period (criterion D2).

## Discussion

Narrow endemic or restricted-range species are conservation targets due to intrinsic biological features [33], [39]. They indicate sites for which there are few spatial options for conservation [7], [9]. Most important, sympatric occurrence of restricted-range species is a valuable indicator of areas of endemism, revealing localized biotas and subjacent evolutionary patterns and biogeographic speciation processes [11], [40], [41]. Alarmingly, restricted-range species tend to be the most poorly represented biodiversity targets in site-selection analyses using coarse-scale biodiversity surrogates [9], [42]. Rarity and lack of adequate data, poor taxonomy and omission errors in conservation analyses make restricted-range species candidates for unrecorded extinction [43], erasing unstudied and relevant indicators of biogeographic patterns and underlying evolutionary mechanisms. The inclusion of ichthyological data in site conservation planning favors the detection of biogeographic, historical patterns of species production and distribution [11]. The fine-grained nature of fish distributions, coupled with high species richness (the most diverse of the traditional vertebrate taxonomic groups), aids in recovering biogeographical patterns and centers of endemism [11], a critical information for conservation [35], [44], [45].

The large percentages endemic species found in Atlantic Forest (overlapping most Atlantic hydrographic regions) or central Brazilian Cerrado (high overlap with Paraná and Tocantins-Araguaia hydrographic regions) agrees with spatial priorities recovered using data on endemism of terrestrial organisms. The two recognized Brazilian biodiversity hotspots, although delimited using vegetational (and not hydrological) boundaries, also harbor high numbers of critical watersheds, indicating that overall patterns of endemism (and, as a result, of threat) may be congruent among different taxonomic groups. Unfortunately, despite the recent accumulation of data for most taxonomic groups, local-scale analyses on patterns of endemism are lacking in the Neotropical Region, and could provide more rigorous tests on emergent biological properties and detailed conservation priorities. However, the general lack of concordance between our results and previous redlisting and local scale priority setting exercises highlights the importance of refined taxonomic and distributional data in conservation analyses.

Our results highlight the importance of refining the scale of biodiversity mapping initiatives to the site level [7], through the compilation and careful revision of voucher-based species distribution data, making proper use of the wealth of data accumulated in zoological collections and literature [6]. The very detection of restricted-range species (and their associated unique habitats) depends on careful revision of taxonomic and distributional data. The compilation of detailed voucher-based distribution data is critically urgent, and should be considered as a crucial step in threat assessments or priority setting exercises in regions that combine high species diversity and high rates of habitat loss, such as most of the Neotropical Region.

A possible caveat of our method of detection of restricted-range species refers to the fact that basic knowledge on Brazilian fish distribution is still incomplete. To avoid underestimation in the number of restricted-range species detected, all currently described species were evaluated. However, there is a possibility that some species were considered as occupying restricted areas due to undersampling of remote or poorly-studied areas. While it is probable that additional data will reveal larger ranges for some of the species, the highest concentration of restricted-range species was detected along the Southern and Southeastern Hydrographic Regions where the fish fauna has been intensively sampled and studied over many decades. Undersampling of remote areas in the large river basins of the Amazon Region inspire more concern, but most restricted-range species from those areas were described in the last couple of decades in the context of comprehensive faunal surveys, which made available large numbers of geographically relevant comparative material. In fact, most of those species were formally described precisely because they were easy to identify and stand out as restricted-range species, while more widely distributed populations still await taxonomic study. Further refinement of our database based on new taxonomic and biogeographic data is both possible and desirable, but we predict that the general pattern resulting from this analysis will be confirmed as knowledge about Neotropical fishes increases.

Our results suggest that the number of threatened freshwater fish species in Brazil is at least four times that currently indicated by global and national red lists. The vast majority of species in our study were never evaluated in IUCN redlist assessments. Formal assessments of known species are likely to increase the number of threatened fishes, as these will include species with larger ranges that are threatened by other factors not examined in the present study, such as exploitation, introduction of alien species and large-scale pollution.

Our findings support the hypothesis that freshwater conservation has been neglected, especially when compared with better studied terrestrial vertebrates, such as birds and mammals [13]. At least in Brazil, freshwater fishes are by far the most threatened vertebrate group, with threat rates similar to those found globally for amphibians [13], also highly dependent on freshwater habitats for reproduction [46]. The low overlap between our results and Brazilian priority areas, based mainly on data on threatened species and terrestrial habitat remnants, indicates that these are not representative of restricted-range species and aquatic ecosystems and organisms in Brazil, or have not been delineated according to watershed limits. Our results reveal previously overlooked sites that will likely be affected by extinction events in the near future, given the continued high rates of habitat loss in most of Brazil.

The general lack of congruence among redlists also corroborates the hypothesis that poor biodiversity knowledge in most of the Neotropical Region results in rates of species loss that may be higher than currently estimated [10]. In fact, extant species with geographically restricted ranges that are not considered endangered or extinct are prime candidates for conservation attention and research [11], [12]. The failure in detecting and highlighting such species and sites would inflate omission errors in biodiversity assessments and further delay crucial information on threat, in areas where irreversible biodiversity losses are predicted in the near future.

Hydropower supplies most Brazilian energy needs [25]. Given that 22% of detected watersheds overlap hydropower dams, and the lack of monitoring studies on restricted-range species in impacted areas, it is highly probable that the 220 species found at these sites are already under high extinction risk. The location of narrow endemic species, along with complementary conservation targets such as maintenance of migratory routes of large, commercially important fish species [47], [48], [49] should be decisive in locating hydropower projects where they will do least damage [1]. However, hydropower plants are still causing harm to entire natural freshwater communities around the globe [10], [50], [51], and their location has not been changed even when causing serious threats to endemic species and their unique habitats [52].

Although threatened species and their ranges are of immediate concern, site-scale conservation assessments should also point endemic faunas that remain free from human impact and may be subject to proactive conservation initiatives [16], especially in large areas such as most Amazonian river catchments or vast wetlands in the Paraguay basin. Although immediate intervention is required in some highly impacted watersheds (such as those under hydropower impact and lacking formal protection), biodiversity data in relatively undisturbed areas in the Neotropical region will uncover fleeing opportunities for conservation [1]. Thus, the detection of impact-free endemic fish faunas is an important information for mid- to long-term conservation action and planning. The inclusion of a large array of focal areas favors both reactive and pro-active conservation, using the best available documented knowledge on species endemism patterns and threat.

Terrestrial and freshwater habitats are naturally linked by biological and physical processes, with freshwater communities directly affected by changes in terrestrial habitat integrity and quality [30]. Entire groups of terrestrial taxa are highly dependent on aquatic habitats in critical stages of their life cycles [1], [46]. Terrestrial anurans with aquatic larvae depend on adjacent and interlinked freshwater and terrestrial habitats, and sites containing intact terrestrial vegetation adjacent to rivers and streams harbor richer anurofaunas [53], highlighting interdependence of freshwater and terrestrial conservation. Thus, conservation planning units and focal areas should include terrestrial and aquatic habitats and species, if they are to protect original biodiversity patterns and processes [1], [10], [54]. The delineation of sites according to natural catchment areas provides a unique possibility for integrating freshwater and terrestrial species data and habitats in ecologically and biogeographically sound conservation initiatives.

Restricted-range species are a critical (although elusive and often overlooked) component of conservation strategies, that should be integrated to other conservation targets in conservation planning initiatives. Given the limitations imposed by knowledge of biodiversity, conservation strategies that combine different targets (such as endemic species, threatened species, keystone species, ecological and evolutionary processes, environmental diversity) are seen as the best options to maximize representation of overall, real biodiversity [55]. A comprehensive strategy for the conservation of freshwater fish species in Brazil needs to address both restricted-range endemic species as well as widespread migratory species that have high commercial value [56]. Efforts need to be done to map and protect areas that are critical for migratory species as well as evaluate the impact of the several dams that have been built or planned in the Brazilian rivers.

A range of different strategies will be essential to preserve Brazilian freshwater biodiversity, and conservation actions may include and be guided by the delineation of biodiversity-relevant catchments, as well as species- or habitat-based actions that reconcile biodiversity protection with the rational use of ecological services in modified drainage-scale ecosystems [10]. Brazil and many other nations have promulgated guidelines for riparian protection, often linked to forestry practices. The Brazilian forestry code explicitly states that micro scale watershed areas (“micro-bacias”) are the mandatory landscape units for legal reserve planning and compensation measures.

In cases where site-based conservation alone would not be effective (for species depending on downstream migration or catchment-regulated flow regimes), larger watersheds including groups of endemic areas as focal areas could be managed through compliance and enforcement of existing legislative codes [54]. The integration with wide-ranging species (both aquatic and terrestrial), requiring action at regional scales, could benefit from the array of smaller units, forming the backbone of larger conservation initiatives. If properly managed and protected, the 540 watersheds detected herein will be decisive in avoiding extinction in the richest aquatic ecosystems on the planet.

## Supporting Information

Table S1 Species occurrence and watershed matrix. Maps and additional data available at http://peixesraros.conservation.org.br.
(0.74 MB XLS)
